# Latent abstraction bridge transformer for generalizable nonintrusive load monitoring

**DOI:** 10.1038/s41598-026-48516-0

**Published:** 2026-04-30

**Authors:** Yuan Liu, Zhengmin Kong, Tao Huang, Yang Yang, Chenjie Song, Qing-Long Han, Boyang Huang

**Affiliations:** 1 School of Electrical Engineering and Automation, Department of Artificial Intelligence and Automation, Wuhan University, Wuhan, 430072 China; 2 College of Science and Engineering, and Centre for AI and Data Science Innovation, James Cook University, Cairns, QLD 4878 Australia; 3 School of Engineering, Swinburne University of Technology, Melbourne, VIC 3122 Australia; 4 Electric Power Research Institute of China Southern Power Grid, Guangzhou, 510700 China; 5 Guangdong Provincial Key Laboratory of Intelligent Measurement and Advanced Metering of Power Grid, Guangzhou, 510700 China

**Keywords:** Beta-variational autoencoder, Soft vector-quantized variational autoencoder, Latent abstraction bridge (LAB), Transformer, Nonintrusive load monitoring, Engineering, Mathematics and computing

## Abstract

Nonintrusive load monitoring (NILM) is an effective approach for energy management that disaggregates the total power measured at the main power inlet into appliance-level power signals. NILM algorithms have achieved remarkable progress in recent years. However, accurately reconstructing appliance-level power signals from unseen, complex, and diverse aggregated data remains a formidable challenge. To address this challenge, this article proposes a novel hybrid load disaggregation model, the Latent Abstraction Bridge (LAB) Transformer, built on a sequence-to-sequence (S2S) framework that integrates a convolutional neural network (CNN) and a Transformer architecture with an embedding-constrained generative network termed LAB. The LAB effectively balances local discrete details and global information by leveraging a soft vector-quantized variational autoencoder (SoftVQ-VAE) and a beta-variational autoencoder (Beta-VAE) to constrain the encoder’s output representations, thereby considerably improving the model’s ability to generalize and discriminate in latent space. Moreover, we use parameter-free linear interpolation to recover the lengths of Beta-VAE output vectors, preserving essential global information while suppressing unnecessary local details, thereby substantially reducing the parameter count. The effectiveness of the proposed model is validated on two datasets: UK-DALE and REFIT. Experimental results indicate that it achieves the best F1 score, while lowering the mean absolute error (MAE) and signal aggregation error (SAE) by 22.8% and 24.7%, respectively, compared to several recent state-of-the-art models.

## Introduction

Nonintrusive load monitoring (NILM), also known as load disaggregation, is a widely adopted technique for tracking and managing electricity usage in residential and industrial environments, thereby improving energy efficiency^1^. The concept was originally introduced by G. W. Hart^2^. NILM seeks to analyze the aggregate electricity usage of a household or building by deploying sensors at the main power entry point. From this aggregate data, the system infers the operational state and energy usage of individual appliances, eliminating the need for intrusive device-level monitoring within the premises. Due to its significance in energy conservation and management^3^, NILM has attracted widespread research interest.

In recent years, the development of NILM has been greatly driven by advances in machine learning and deep learning. Conventional machine learning approaches are generally classified as supervised or unsupervised. Supervised techniques, including k-nearest neighbors^4^, support vector machines^5^, and artificial neural networks^6^, utilize labeled datasets to identify appliance states or estimate their power usage. These methods typically achieve promising performance when sufficient labeled data are available. Unsupervised approaches, such as the hidden Markov model and its extensions^7^, as well as clustering techniques like k-means^8^, operate without labeled appliance-level data. Instead, they attempt to identify underlying patterns or latent components to disaggregate the total consumption into individual appliance contributions, demonstrating strong self-learning capabilities and an aptitude for discovering hidden structures. However, the appliance signals reconstructed by traditional machine learning methods are prone to significant interference due to complex overlapping power signatures^9^.

Compared to the aforementioned traditional methods, deep learning methods demonstrate superior capabilities in capturing the complex features of multi-state appliances^10^. The use of three deep neural network architectures for appliance-level load disaggregation was first investigated by Kelly and Knottenbelt^11^. Their work introduced the long short-term memory (LSTM) network, the denoising autoencoder (DAE), and a regression model designed to jointly estimate the start and end times and the mean power usage of individual appliance activations. These methods inspired further exploration into sequence-to-sequence (S2S) model designs. A novel efficient learning model, termed sequence-to-point (S2P)^12^, was proposed based on the S2S learning model. However, the S2P model produces only one output value per forward pass, resulting in high computational cost during inference^13^. Therefore, greater emphasis has been placed on the design of S2S models. A subtask-gated network (SGN)^14^ was proposed based on the S2S model that integrates a primary regression model with an auxiliary on/off classification task, thereby achieving higher disaggregation accuracy. The S2S model was enhanced by Bousbiat *et al.*^15^ through the incorporation of an imaging block that transforms the input sequence into an image.

Although these deep learning methods have enhanced the ability to extract appliance features, their performance in NILM remains hindered by severe misclassification and missed-detection issues due to the intricate similarities among appliance power-consumption patterns^16^. In light of this, Transformer architectures^17^, with their powerful ability to model sequential data, have attracted increasing interest from researchers working on NILM tasks. A model based on Bidirectional Encoder Representations from Transformers (BERT)^18^ was developed^19^, adopting an S2S learning framework to enhance load disaggregation performance. A Transformer-based model^20^ similar to the SGN was introduced and shown to achieve competitive disaggregation accuracy and generalization, with additional exploration of transfer learning across datasets.

Furthermore, generative models, which exhibit strong capabilities in modeling intricate data structures and generating realistic, high-fidelity outputs^21^, have recently re-emerged as a research focus. An adversarial autoencoder was proposed^22^ to improve the generalization capacity of load disaggregation. A generative adversarial network (GAN)^23^ was introduced, aiming to enhance both the precision and robustness of disaggregation. While GANs are known for generating high-quality samples, variational autoencoders (VAEs) offer the added advantage of learning meaningful latent structures through an encoder–decoder architecture grounded in variational inference^24^. A model that combines CNNs with VAEs^25^ was investigated, enabling effective capture of general features. An S2S learning-based model^9^ that utilizes a Beta-VAE and a novel channel attention mechanism to enhance load disaggregation performance, targeting improved accuracy under complex load conditions.

Although NILM techniques based on advanced artificial intelligence (AI) models have advanced rapidly, a significant challenge remains in their application to unseen buildings due to limited generalization. More specifically, reconstructing accurate appliance-level power signals from complex, diverse aggregated power data remains a difficult task in unseen scenarios. In particular, the absence of accurate and robust encoded latent representations for unseen buildings often leads to misclassifications or missed detections. Therefore, developing a model that learns generalizable, discriminative latent representations from aggregated power data is crucial.

To overcome the challenge, a novel architecture is proposed that integrates a scoring network with a soft vector-quantized variational autoencoder (SoftVQ-VAE)^26,27^, and Beta-VAE, and incorporates this into a Transformer framework for load disaggregation tasks. In the proposed model, a sequence encoder, a latent abstraction bridge (LAB) designed to enhance the generalization capability of the encoded vectors, and a decoder are constructed. The encoder maps the aggregated power data into a latent space and encodes it as a sequence of vectors. The LAB constructs a codebook and performs a constrained representation of the vector sequence through soft quantization. Additionally, a Beta-VAE is incorporated along with a scoring network to capture global information and mitigate potential fluctuations in the reconstructed signals. Finally, the decoder reconstructs appliance-level power signals based on the representations. We conduct comprehensive comparisons between the proposed model and several state-of-the-art models on two low-frequency real-world datasets. The results demonstrate that the proposed model achieves more accurate detection of appliance on/off events and more precise power signal disaggregation, exhibiting significantly improved generalization performance. The main contributions of the work are summarized as follows:

- A novel hybrid load disaggregation model, LAB Transformer, is proposed that combines CNN and Transformer architectures with an embedding-constrained generative network. This model is designed to accurately reconstruct appliance-level power signals by effectively balancing multi-scale information. Experimental evaluations on two low-frequency real-world datasets demonstrate that the proposed model achieves superior generalization performance compared to state-of-the-art algorithms, achieving the best F1 score while reducing mean absolute error (MAE) and signal aggregation error (SAE) by 22.8% and 24.7%, respectively.
- A LAB is introduced to further constrain and abstract the encoded representations from the encoder, thereby enhancing the model’s feature learning capability. More specifically, it is a scoring network built upon SoftVQ-VAE and Beta-VAE, designed to balance discrete representations and global information. As a result, the LAB significantly improves the generalizability and discriminative power of the model’s latent representations.
- Parameter-free linear interpolation is adopted to reduce network complexity. This modification preserves global patterns while suppressing unnecessary local details that would otherwise require numerous additional parameters. In contrast, traditional Beta-VAEs typically utilize fully connected or transposed convolutional layers to recover the vector length, which significantly increases parameter count.

To provide a clear overview of the differences between the proposed model and existing algorithms, Table 1 summarizes the key characteristics of each model.

The paper is organized as follows. Section 2 reviews the background of NILM and defines the problem. Section 3 introduces the proposed approach and explains its architecture. Section 4 describes the datasets together with the experimental setup. Section 5 reports and analyzes the results in detail. Finally, Section 6 summarizes the work and outlines directions for future study.

**Table 1:** Comparison of key characteristics among models.

Features					
Models	LAB (Ours)	DAE^11^	SGN^14^	CM^20^	IECA^9^
**S2S**	✓	✓	✓	✓	✓
**Generative Model**	✓				✓
**Lightweight Design**	✓			✓	✓
**Bridge-like Structure**	✓				✓
**Latent Abstraction Bridge**	✓				
**Local Detail Suppression**	✓				

## Background and problem statement

### Load disaggregation

Load disaggregation aims to decompose a building’s total electricity usage into the consumption profiles of individual appliances. At time *t*, the aggregate power reading, denoted by $y_t$, can be expressed as the sum of the power usage from *N* appliances together with noise, as shown below:

1 $$\begin{aligned} y_t = \sum _{i=1}^{N}x_{i,t} + \epsilon _t. \end{aligned}$$

In this formulation, $x_{i,t}$ corresponds to the power consumed by appliance *i* at time *t*, while $\epsilon _t$ accounts for measurement noise or unmodeled factors. The goal of load disaggregation is to recover the set $\{x_{i,t}\}_{i=1}^N$ from the observed aggregate signal $y_t$.

### S2S learning

In S2S learning for NILM, the objective is to map a sequence of aggregated power measurements to a corresponding sequence of estimates of appliance-level power consumption. Specifically, given an input sequence of aggregated power readings:

2 $$\begin{aligned} Y = [y_t, y_{t+1}, \cdots , y_{t+w-1}] \end{aligned}$$

where *w* defines the window size, the model aims to predict the corresponding output sequence:

3 $$\begin{aligned} X = [x_t, x_{t+1}, \cdots , x_{t+w-1}] \end{aligned}$$

for the target appliance. This process can be formally expressed as learning a mapping function $f_{\theta }$, parameterized by $\theta$, where $\theta$ denotes the set of learnable model parameters (e.g., weights and biases). The mapping produces the estimated power consumption sequence:

4 $$\begin{aligned} \hat{X} = f_{\theta }(Y) \end{aligned}$$

where $\hat{X}$ denotes the estimated power consumption sequence of the target appliance. The goal of the S2S model is to minimize the discrepancy between $\hat{X}$ and the ground truth *X*, thereby enabling accurate disaggregation from aggregate signals.

## LAB transformer architecture

### Latent abstraction bridge module

**Fig. 1:**
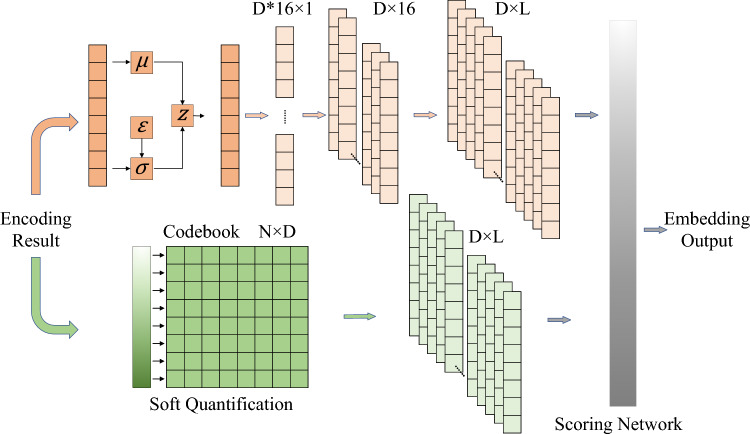
The structure of the latent abstract bridge.

This study introduces a new module, called the *latent abstraction bridge* (LAB), which imposes additional constraints on the encoder’s representations to enhance the generalization and discriminative power of the latent features. LAB integrates SoftVQ-VAE and Beta-VAE within a unified scoring network framework. Specifically, SoftVQ-VAE focuses on capturing fine-grained discrete representations, while Beta-VAE is designed to extract global features and suppress redundant local details. The outputs from these two components are fed into a scoring network that assigns weights to each representation and fuses them into a new embedding vector. This fused latent representation is subsequently passed to the decoder to reconstruct appliance-level power signals. The architecture of LAB is illustrated in Fig. 1, and its components are described in detail below. A detailed forward propagation process of the LAB module is provided in the supplementary material, Section “LAB Forward Procedure”.

#### Beta-VAE

Beta-VAE builds on the standard VAE^24^, which learns probabilistic latent variables from the input and uses them to regenerate the data in its original form. To encourage the learning of disentangled and interpretable latent factors, Beta-VAE modifies the conventional VAE loss by incorporating a weighting parameter $\beta$ into the Kullback–Leibler (KL) divergence term:

5 $$\begin{aligned} \begin{aligned} \mathscr {L}_{\text {Beta-VAE}} =&\mathbb {E}_{q_{\phi }(z|x)} [\log p_{\theta }(x|z)] \\ &- \beta \cdot D_\mathrm {{KL}}(q_{\phi }(z|x)||p(z)). \end{aligned} \end{aligned}$$

Here, *p*(*z*) represents the prior distribution over the latent variables, usually chosen as a standard Gaussian $\mathscr {N}(0,I)$. The decoder network, parameterized by $\theta$, models the likelihood function $p_{\theta }(x|z)$, while the encoder $q_{\phi }(z|x)$, with parameters $\phi$, approximates the posterior. In this work, $\beta$ is set to 0.001 to emphasize reconstruction accuracy.

Within the proposed model, Beta-VAE plays a crucial role in enhancing the global contextual representation of latent variables, thereby mitigating potential fluctuations during signal reconstruction. To achieve this, a fully connected layer is first used to project the processed vector into a shorter representation. The vector is then upsampled to its original length using parameter-free linear interpolation. This method effectively preserves global information while suppressing redundant local details, significantly reducing the number of parameters required to capture such nuances.

#### SoftVQ-VAE

The SoftVQ-VAE^27^ is employed to learn compact discrete representations while maintaining full differentiability. Unlike the standard vector-quantized variational autoencoder (VQ-VAE)^26^, which performs hard assignments by mapping each input to the nearest codebook vector, SoftVQ-VAE introduces a soft assignment mechanism that represents the encoder output as a weighted combination of multiple codebook entries.

Given an input embedding $z_e \in \mathbb {R}^D$ produced by the encoder, the similarity between $z_e$ and each codebook vector $e_k$ is computed, typically using negative squared Euclidean distance or dot product. These similarity scores are transformed into a probability distribution over the codebook entries via a softmax function with temperature $\tau$:

6 $$\begin{aligned} w_k=\frac{\exp (s_k/\tau )}{\sum _j \exp (s_j/\tau )}. \end{aligned}$$

Here, $s_k$ denotes the similarity between $z_e$ and $e_k$, and $w_k$ is the corresponding soft assignment weight. A smaller $\tau$ results in sharper assignments that approximate hard quantization, while a larger $\tau$ produces smoother distributions across the codebook entries. The quantized embedding $z_q$ is computed as follows:

7 $$\begin{aligned} z_q=\sum _{k}w_ke_k. \end{aligned}$$

This soft quantization allows the model to remain fully differentiable. During training, the objective consists of a reconstruction loss that encourages accurate reconstruction of the input from the quantized embedding, and a KL regularization term that promotes a soft assignment distribution close to a desired prior (e.g., uniform), thereby ensuring effective utilization of the codebook.

In this work, a discrete codebook is employed to impose constraints and enable deeper abstraction of the encoder outputs. Specifically, the encoded representations are re-expressed exclusively in terms of vectors from the codebook, thereby enforcing a structured, limited representational space. The use of soft quantization enriches the codebook’s representational capacity and enhances the model’s feature-extraction capabilities. SoftVQ-VAE is particularly effective at capturing fine-grained details in reconstructed signals, making it well-suited for modeling complex power-signal segments, especially those exhibiting frequent and abrupt changes.

#### Scoring network

**Fig. 2:**
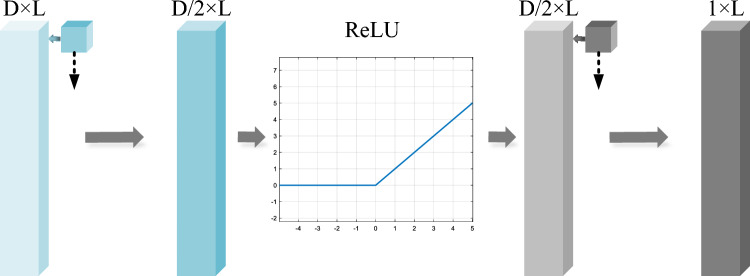
The structure of the scoring network.

The scoring network is designed to evaluate the latent representations extracted from both the SoftVQ-VAE and Beta-VAE branches and to assign a weight to each. The detailed architecture of the scoring network is illustrated in Fig. 2. A softmax function is then applied across the two branches to obtain normalized weights, which are used to fuse the latent outputs into a single representation for each time step.

This design provides an efficient and flexible mechanism for dynamically weighting the latent representations derived from distinct encoding branches. By employing $1 \times 1$ convolutions, the network performs per-position channel-wise projections without altering the temporal resolution. This allows the capture of feature importance at each time step with minimal computational overhead. This architecture enables efficient per-timestep weighting of latent features. Furthermore, the use of a shared scoring mechanism for both branches ensures consistent evaluation criteria, thereby facilitating more stable and adaptive fusion of latent representations.

It is worth noting that the scoring network is specifically designed to regulate the contribution of complementary low-scale information from the Beta-VAE branch. Due to the interpolation operation, the Beta-VAE branch inevitably discards fine-grained nonlinear details. However, the information preserved is exactly the coarse-scale information required, i.e., the global characteristics across the entire sliding window. In contrast, the SoftVQ-VAE branch focuses on capturing high-scale details, namely the precise short-term signatures of appliance operation.

This dual-branch design is motivated by the observation that, when using only the SoftVQ-VAE branch for disaggregation, the reconstructed power signals sometimes exhibit fluctuations. Incorporating coarse-scale contextual information effectively mitigates this issue and significantly improves disaggregation performance. The ablation study results validate the effectiveness of the multi-scale design.

### The overall structure of LAB transformer

**Fig. 3:**
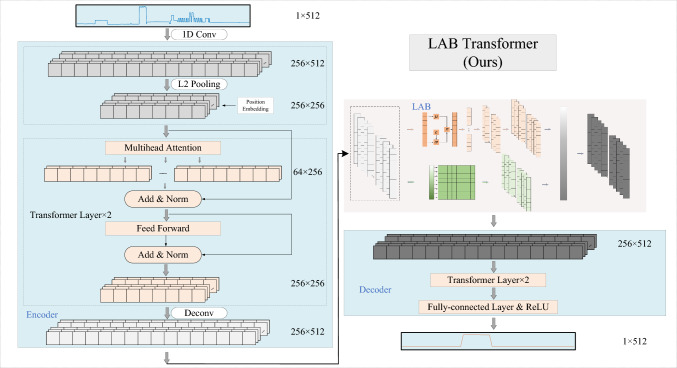
Overall structure of the LAB Transformer.

The proposed model consists of an encoder, a LAB module, and a decoder, as illustrated in Fig. 3. The encoder first extracts features and compresses the sequence length of the input $Y \in \mathbb {R}^{B \times 1 \times L}$ using a 1D convolutional layer with 256 kernels, followed by an $L_2$ pooling layer. This process is formulated as:

8 $$\begin{aligned} Y_{e} = \text {L2Pool}(\text {Conv1D}(Y)). \end{aligned}$$

Here, $\text {Conv1D}(\cdot )$ applies convolutional filters to extract features, and $\text {L2Pool}(\cdot )$ performs downsampling by computing the $L_2$-norm within each pooling window, resulting in output $Y_e \in \mathbb {R}^{B \times D \times L'}$. This strategy enables efficient feature extraction while reducing the sequence length, thereby lowering the computational complexity.

After feature compression, positional encoding is added to preserve temporal order information, as expressed by:

9 $$\begin{aligned} Y_p = Y_e + E_{\text {pos}}. \end{aligned}$$

The sequence $Y_p$ is then passed through layer normalization and dropout for regularization and stabilization:

10 $$\begin{aligned} Y_{n} = \text {Dropout}(\text {LN}(Y_p)). \end{aligned}$$

Here, $\text {LN}(\cdot )$ denotes layer normalization, and $\text {Dropout}(\cdot )$ refers to dropout regularization. Given the input tensor $Y_p$, the layer normalization operation is defined as follows:

11a $$\begin{aligned}&\hat{Y}_{p_{b,:,l}}=\frac{{Y}_{p_{b,:,l}}-{\mu }_{b,l}}{\sigma _{b,l}} \cdot g + b_0, \end{aligned}$$

11b $$\begin{aligned}&{\mu }_{b,l} = \frac{1}{D} \sum ^{D}_{i=1}Y_{p_{b,i,l}}, \end{aligned}$$

11c $$\begin{aligned}&{\sigma _{b,l}}=\sqrt{\frac{1}{D} \sum ^{D}_{i=1}{(Y_{p_{b,i,l}}-\mu _{b,l})}^2+\epsilon }. \end{aligned}$$

Here, $\hat{Y}_{p_{b,:,l}}$ denotes the normalized output at time step *l* for batch *b*, while ${\mu }_{b,l}$ and ${\sigma }_{b,l}$ represent the mean and standard deviation computed across the feature dimension, respectively. The parameters *g* and $b_0$ are learnable affine transformation parameters of dimension *D*, and $\epsilon$ is a small constant added for numerical stability, set to $1 \times 10^{-5}$.

The normalized sequence $Y_{n}$ is subsequently passed into a Transformer encoder to model long-range dependencies and interactions between time steps. Multi-head self-attention (MHSA) is then applied to capture contextual relationships. For each attention head, the input $Y_n \in \mathbb {R}^{B \times D \times L'}$ is linearly projected into queries *Q*, keys *K*, and values *V* as follows:

12 $$\begin{aligned} Q = Y_nW^{Q},\quad K = Y_nW^{K},\quad V = Y_nW^{V}. \end{aligned}$$

Here, $W^Q$, $W^K$, and $W^V$ are learnable matrices of size $\mathbb {R}^{D \times D_h}$, where $D_h = D/H$ and $H = 4$ is the number of attention heads. The scaled dot-product attention is computed as:

13 $$\begin{aligned} \text {Attention}(Q, K, V) = \text {Softmax}\left( \frac{QK^\text {T}}{\sqrt{D_h}}\right) V. \end{aligned}$$

Outputs from all heads are concatenated and projected back to the original dimension. A residual connection and layer normalization are applied as follows:

14 $$\begin{aligned} Y_\text {attn} = \text {LN}(Y_n + \text {Dropout}(\text {MHSA}(Y_n))) \end{aligned}$$

where $\text {MHSA}(\cdot )$ represents the multi-head self-attention computation.

This is followed by a position-wise feed-forward network (FFN) composed of two linear layers and a Gaussian error linear unit (GELU) activation function:

15 $$\begin{aligned} \text {FFN}(x) = \text {GELU}(xW_1 + b_1)W_2 + b_2 \end{aligned}$$

where $W_1 \in \mathbb {R}^{D \times ff_{dim}}$ and $W_2 \in \mathbb {R}^{ff_{dim} \times D}$. Another residual connection and normalization step are applied:

16 $$\begin{aligned} Y_{\text {ffn}} = \text {LN}(Y_{\text {attn}} + \text {Dropout}(\text {FFN}(Y_{\text {attn}}))). \end{aligned}$$

The final output $Y_{\text {ffn}}$ is passed through a dropout layer and a transposed convolutional layer for upsampling, restoring the sequence length to *L* and producing the final encoded output $Y_{\text {out}}$.

The decoder adopts a structure similar to that of the encoder, but operates with a reduced latent dimension *D*. It concludes with an output layer comprising two fully connected layers with a ReLU activation in between, which reconstructs the appliance-level power signals. The LAB module operates on the encoder’s encoded outputs, performing deep abstraction and imposing representational constraints to produce refined latent vectors. These vectors are then forwarded to the decoder for signal reconstruction.

### Loss function

The loss function of the proposed model consists of three components. First, the reconstruction loss is computed as the mean squared error (MSE) between the reconstructed appliance-level power signals and the ground-truth signals, thereby ensuring accurate signal recovery. Second, a KL divergence loss from the Beta-VAE module is incorporated to regularize the latent space. The strength of this regularization is controlled by the hyperparameter $\beta$, which is set to 0.001.

This KL term enforces the approximate posterior *q*(*z*|*x*) to stay close to a factorized prior $p(z)=\mathscr {N}(0, I)$.

Third, a KL divergence loss^27^ is applied to the empirical codebook usage distribution, promoting similarity to a uniform distribution and thereby encouraging balanced usage of codebook entries. This term is weighted by a factor $\gamma$, set to 0.25.

These components are jointly optimized to balance reconstruction fidelity, disentanglement of latent representations, and efficient codebook utilization. The total loss function is defined as $\mathscr {L}_{\text {total}}=\mathscr {L}_{\text {rec}}+\beta \cdot \mathscr {L}_{\text {KL}}+\gamma \cdot \mathscr {L}_{\text {codebook}}$ where

17a $$\begin{aligned}&\mathscr {L}_{\text {rec}}=\frac{1}{BT}\sum ^{B}_{b=1}\sum ^{T}_{t=1}||x_{b,t} - \hat{x}_{b,t}||^2, \end{aligned}$$

17b $$\begin{aligned}&\mathscr {L}_{\text {KL}}=D_{\text {KL}}[q(z|x)||p(z)], \end{aligned}$$

17c $$\begin{aligned}&\mathscr {L}_{\text {codebook}}=D_{\text {KL}}[q(e)||U]. \end{aligned}$$

Here, *T* denotes the sequence length, while $x_{b,t}$ and $\hat{x}_{b,t}$ represent the ground truth and the reconstructed outputs, respectively. The term *q*(*e*) denotes the empirical distribution of codebook usage, and *U* is the uniform distribution. The distribution *q*(*e*) is estimated using the soft assignment probabilities produced by the SoftVQ-VAE. Specifically, let $w_{b,t,k}$ denote the soft assignment probability of the *k*-th codebook vector for the token at temporal position *t* in the *b*-th sample of a minibatch. The empirical usage distribution is then computed as:

18 $$\begin{aligned} q_k=\frac{1}{BT}\sum _{b=1}^{B}\sum _{t=1}^{T}w_{b,t,k}. \end{aligned}$$

Then $\mathscr {L}_{\text {codebook}}$ is defined as:

19 $$\begin{aligned} \mathscr {L}_{\text {codebook}}=D_{\text {KL}}[q(e)||U]=\sum _k^{N_e}q_k\log \frac{q_k}{1/N_e}, \end{aligned}$$

where $N_e$ denotes the number of codebook vectors. During backpropagation, $\mathscr {L}_{\text {codebook}}$ encourages the empirical usage frequency of each codebook vector to approach a uniform distribution, thereby mitigating codebook collapse.

## Experimental setup

### Dataset

The public datasets UK-DALE^28^ and REFIT^29^ are used to train and evaluate the proposed model. UK-DALE contains both aggregate mains power measurements and sub-metered appliance-level power data, collected from five typical UK households at a sampling frequency of 1/6 Hz. Owing to its high data quality, UK-DALE has become a widely adopted benchmark in NILM. The REFIT dataset provides detailed residential energy consumption data from 20 UK households, recorded between 2013 and 2015, sampled at 1/8 Hz. Given its wide coverage of household appliances and consistent data quality, REFIT is also frequently used in NILM research. In this study, the UK-DALE dataset is downsampled to match REFIT, and all experiments on both datasets are conducted at a unified sampling frequency of 1/8 Hz.

### Data preprocessing

Two primary preprocessing steps are performed to ensure data quality and consistency. First, the indices of the aggregate mains data and the sub-metered appliance data are aligned, and any missing values are removed to ensure temporal consistency across data sources. Second, Z-score normalization is applied independently to the aggregate data and the target appliance data in order to standardize their distributions. Z-score normalization is defined as follows:

20 $$\begin{aligned} Y_{\text {Z}} = \frac{Y-\mu }{\sigma } \end{aligned}$$

where *Y* denotes the original input value, $\mu$ represents the mean of the series, and $\sigma$ denotes the standard deviation.

### Metrics

To enable comparison with existing load disaggregation models, three evaluation metrics are employed: MAE, SAE, and F1 score. MAE quantifies the average magnitude of the error between the predicted and ground-truth values. It measures overall prediction accuracy in terms of absolute deviation and is defined as:

21 $$\begin{aligned} \text {MAE} = \frac{1}{T} \sum ^T_{t=1} |x_t - \hat{x}_t| \end{aligned}$$

where $x_t$ and $\hat{x}_t$ denote the ground truth and predicted values at time *t*, respectively.

SAE measures the average per-window aggregate error between the predicted and ground truth values and is defined as:

22 $$\begin{aligned} \text {SAE} = \frac{1}{N_W} \sum ^{N_W}_{i=1} \frac{\left| \sum ^T_{t=1} \hat{x}_{i,t} - \sum ^T_{t=1} x_{i,t}\right| }{T}. \end{aligned}$$

Here, $N_W$ denotes the number of windows, *T* represents the window length, and $x_{i,t}$ and $\hat{x}_{i,t}$ denote the ground truth and predicted values at time *t* within window *i*, respectively.

In NILM, the F1 score measures the accuracy with which the model identifies the on/off states of appliances. It is defined as:

23 $$\begin{aligned} F_1 = \frac{2\text {TP}}{2\text {TP}+\text {FP}+\text {FN}}. \end{aligned}$$

Here, $F_1$ is computed from the numbers of true positives (TP), false positives (FP), and false negatives (FN).

In this work, a positive sample refers to a time step at which the target appliance is operating, i.e., its power consumption exceeds the predefined activation threshold. Conversely, a negative sample denotes a time step at which the appliance remains off, with its power consumption falling below the activation threshold. The activation thresholds for kettle, fridge, dishwasher, washing machine, and microwave are set to 1000 W, 50 W, 10 W, 20 W, and 200 W, respectively, as in^11^.

### Scenario configuration

In Scenario 1, House 1 and House 5 from the UK-DALE dataset are used for training, while House 2 is used for testing. Houses 3 and 4 are excluded because they lack the main meter data required for model input. Specifically, approximately 1.5 years of data from House 1 and 0.5 years from House 5 are used for training, while 0.5 years of data from House 2 are used for testing. In the REFIT dataset, not all houses contain the target appliances considered in this study. Therefore, house selection is restricted to those that include the required appliances^9,20^. Table 2 lists the house used in Scenario 2. The training set for each appliance contains more than two years of data in total. Scenario 3 corresponds to a cross-dataset evaluation setting, where models are trained on the REFIT dataset and evaluated on house 2 of the UK-DALE dataset. For all scenarios, the data are split into training and validation sets in an 8:2 ratio.

### Hyperparameter configuration

Table 3a and Table 3b list the detailed hyperparameter configurations used for training each model. The abbreviations KT, FR, DW, WM, and MW denote Kettle, Fridge, Dishwasher, Washing Machine, and Microwave, respectively. Specifically, B denotes the batch size, Win refers to the length of the input sliding window, S is the stride, Opt represents the optimizer, and LR is the learning rate. The proposed model employs a sliding window of length 512 with a stride of 64, while accounting for both the codebook size and the complete operating cycle of each appliance^30^. The model is optimized using the Adam optimizer with a learning rate of 0.0001. The $\tau$ values of the SoftVQ-VAE components for the kettle, fridge, dishwasher, washing machine, and microwave are set to 2.0, 1.0, 0.1, 0.8, and 1.0, respectively. For the remaining four baseline models, the hyperparameter settings are adopted from their respective original publications, with necessary adaptations applied when certain parameters were not explicitly specified.

Because the model uses the Transformer architecture, it is initialized with pretrained weights to accelerate convergence. All models are trained for 100 epochs, and early stopping is applied to prevent overfitting.

**Table 2:** Houses used for training and testing.

Appliance	Scenario 2: REFIT	
	Training	Testing
Kettle	4, 5, 7, 8, 9, 12, 13, 19	2
Fridge	2, 5, 9, 12	14
Dishwasher	1, 2, 5, 7, 9, 13, 17, 19	6
Washing Machine	1, 2, 5, 7, 9, 15, 16, 17, 18	8
Microwave	2, 5, 9, 10, 12, 14, 17, 19	4

**Table 3:** Parameters of experiment.

Parameter	LAB	SGN	CM							
	For All Appliances									
(a) Parameters of LAB (Ours), SGN, and CM										
B	32	64	32							
Win	512	599	480							
S	64	32	64							
Opt	Adam	Adam	Adam							
LR	0.0001	0.0001	0.0001							

Appliance	DAE					IECA				
	B	Win	S	Opt	LR	B	Win	S	Opt	LR
KT	64	256	16	Adam	0.0001	150	1024	64	RMSProp	0.001
FR	64	512	16	Adam	0.0001	150	1024	64	RMSProp	0.001
DW	64	512	16	Adam	0.0001	32	1024	256	RMSProp	0.001
WM	64	512	16	Adam	0.0001	32	1024	256	RMSProp	0.001
MW	64	256	16	Adam	0.0001	150	1024	64	RMSProp	0.001

### Hardware and software support

The experimental setup was run on a workstation equipped with an Intel Core i9-9900K CPU, an NVIDIA RTX 4090 graphics card, and 64 GB of memory. The software environment was configured with Python 3.8.19 and PyTorch 2.3.0.

## Results and analysis

In this section, the proposed model is compared and analyzed against four baseline models: DAE^11^, SGN^14^, CM^20^, and IECA^9^. DAE and SGN are well-established baseline methods for NILM, whereas CM and IECA are recent state-of-the-art approaches. Therefore, they are selected as the benchmark models for comparison.

The evaluation considers both event detection and disaggregation performance, using F1 score, MAE, and SAE as performance metrics. Higher F1 scores reflect better performance, whereas lower MAE and SAE values are desirable. Table 4 reports the results for Scenario 1, Table 5 presents those for Scenario 2, and Table 6 summarizes the results for Scenario 3. Bold values in the tables indicate the best performance for each metric. The detailed performance ranges of the models are provided in Tables S2 and S3 of the supplementary material.

### Comparison experiment in scenario 1

In Scenario 1, the proposed model consistently achieves the best average performance across all appliances. Specifically, it improves the F1 score by 53.6% compared to DAE and SGN, and by 46.2% compared to IECA, another generative model. Even compared with CM, which adopts a similar Transformer-based architecture, the proposed model delivers marginal yet consistent improvements. Overall, the model significantly reduces the average MAE and SAE across all appliances by 30% to 50% compared to baseline models.

For the kettle, CM achieves the highest F1 score, while the proposed model ranks second, trailing by only 2.0%. However, the proposed model demonstrates consistent improvements in both MAE and SAE. This performance gain is attributed to the strong generalization capabilities introduced by the LAB-based multi-scale feature fusion.

Among all appliances, the fridge exhibits the most consistent performance across different models. This stability is likely due to its continuous operation, which yields more positive samples in the aggregate power signal. Moreover, the limited number of households in the UK-DALE reduces the learning complexity for this appliance. Although the proposed model ranks second in terms of MAE and SAE, with only marginal differences from the best-performing model, it achieves the highest F1 score.

For appliances such as the dishwasher, washing machine, and microwave, the power consumption patterns are inherently more complex. Additionally, their irregular and infrequent use yields fewer positive samples, and their signals are more susceptible to interference from other appliances. These factors contribute to greater performance variability across models. Nonetheless, this variability highlights the strength of the proposed model in effectively capturing appliance-specific characteristics and achieving accurate disaggregation, even under challenging conditions involving complex appliances and limited training data.

The proposed model not only outperforms CNN- or fully connected-based models, such as DAE, SGN, and IECA, but also achieves clear performance gains over CM, which shares a Transformer backbone. These results demonstrate that the latent representations refined by LAB, through constraint and re-encoding, and the integration of discrete fine-grained features and global contextual information enable the model to capture more essential and abstract characteristics. Consequently, this yields more generalizable and discriminative representations that improve performance across diverse appliances.

**Table 4:** Result on the evaluation metrics for the UK-DALE Dataset Scenario 1.

Metric	Model	Kettle	Fridge	Dishwasher	Washing Machine	Microwave	Average
F1 score	**LAB (Ours)**	0.9477 **(2nd)**	**0.9363**	**0.6463**	0.8904 **(2nd)**	**0.7479**	**0.8337**
	DAE	0.7722	0.8231	0.6045	0.1701	0.3425	0.5425
	SGN	0.9138	0.8901	0.1581	0.1774	0.3599	0.4999
	CM	**0.9674**	0.9245	0.5078	**0.9079**	0.7004	0.8016
	IECA	0.9232	0.9154	0.3576	0.2696	0.3848	0.5701
MAE (W)	**LAB (Ours)**	**5.1915**	12.9306 **(2nd)**	**20.7601**	**8.4301**	9.0393 **(2nd)**	**11.2703**
	DAE	10.5632	20.0500	27.6833	17.1199	12.9516	17.6736
	SGN	18.2309	19.7899	28.9315	15.4019	20.2617	20.5232
	CM	5.9296	**12.9100**	25.4395	20.5393	**7.1641**	14.3965
	IECA	11.1089	14.2539	29.1198	15.8642	13.9142	16.8486
SAE (W)	**LAB (Ours)**	**4.0701**	8.6223 **(2nd)**	**17.6853**	**7.4299**	8.2430 **(2nd)**	**9.2101**
	DAE	8.5737	11.6472	24.7478	15.5447	9.8663	14.0759
	SGN	13.6782	11.5447	24.4358	12.4185	17.1302	15.8415
	CM	4.4350	**8.1057**	22.0499	19.5912	**6.1966**	12.0757
	IECA	7.8148	8.9433	24.6252	13.2243	9.6805	12.8576

**Table 5:** Result on the evaluation metrics for the REFIT Dataset Scenario 2.

Metric	Model	Kettle	Fridge	Dishwasher	Washing Machine	Microwave	Average
F1 score	**LAB (Ours)**	**0.7221**	**0.8565**	**0.7248**	**0.7862**	**0.6071**	**0.7393**
	DAE	0.2355	0.7963	0.4117	0.3090	0.4368	0.4367
	SGN	0.5047	0.8084	0.1061	0.5559	0.5576	0.5065
	CM	0.7099	0.8445	0.6753	0.7740	0.5341	0.7076
	IECA	0.4464	0.8543	0.2750	0.3578	0.5508	0.4969
MAE (W)	**LAB (Ours)**	**14.8452**	**11.1772**	**3.5010**	**11.3681**	7.5772 **(2nd)**	**9.6937**
	DAE	29.0426	19.3588	5.9585	30.4476	9.2100	18.8035
	SGN	34.3862	24.6471	18.7677	30.3073	10.0510	23.6319
	CM	17.4898	13.7986	6.5892	19.4635	**6.5208**	12.7724
	IECA	29.9593	13.9186	6.9235	25.8461	11.0052	17.5305
SAE (W)	**LAB (Ours)**	**10.4854**	**7.4118**	**3.0147**	**9.2399**	6.3514 **(2nd)**	**7.3006**
	DAE	21.1876	9.6703	4.5728	27.3870	7.1610	13.9957
	SGN	24.9018	13.7903	16.4024	26.1927	8.2823	17.9139
	CM	13.1036	8.4300	5.9889	15.9415	**5.7545**	9.8437
	IECA	19.6731	7.4784	3.8611	19.6197	7.1164	11.5497

**Table 6:** Result on the evaluation metrics for scenario 3.

Metric	Model	Kettle	Fridge	Dishwasher	Washing Machine	Microwave	Average
F1 score	**LAB (Ours)**	0.7570 **(2nd)**	**0.9038**	0.6248 **(2nd)**	**0.8257**	0.7060 **(2nd)**	**0.7635**
	DAE	0.6017	0.7902	0.6060	0.2446	0.5693	0.5623
	SGN	**0.8193**	0.8515	0.1703	0.6355	0.6802	0.6314
	CM	0.7019	0.8760	**0.6678**	0.7701	**0.7471**	0.7526
	IECA	0.7366	0.8915	0.5146	0.4366	0.5839	0.6326
MAE (W)	**LAB (Ours)**	**13.9426**	19.8305	13.2216	**6.2806**	**4.4167**	**11.5384**
	DAE	22.4922	21.9165	17.3408	13.8758	7.7865	16.6824
	SGN	26.7196	24.3347	21.8813	16.8502	10.0201	19.9612
	CM	15.7811	18.2281	**9.8049**	12.1913	5.5395	12.3090
	IECA	21.0398	**15.8447**	10.8200	12.0802	8.9100	13.7389
SAE (W)	**LAB (Ours)**	**11.0804**	9.4954	11.2155	**5.2027**	**3.4027**	**8.0793**
	DAE	18.1997	9.3675	14.1244	13.0783	5.9880	12.1516
	SGN	19.7396	10.1922	17.0006	15.8282	7.8345	14.1190
	CM	13.2161	8.3116	7.0027	11.3799	4.1270	8.8075
	IECA	14.9025	**7.9903**	**6.3154**	9.3723	6.0364	8.9234

### Comparison experiment in Scenario 2

Scenario 2 is conducted on the REFIT dataset, which contains more households than UK-DALE, thereby providing a richer, more diverse data environment. Under this scenario, the proposed model demonstrates even stronger performance, indicating that its improvement in generalization capability is both consistent and robust. When exposed to larger and more complex data distributions, the model continues to accurately extract deep, meaningful features from power-consumption signals.

For the kettle and fridge, which are used more frequently and contain more positive samples, the proposed model achieves noticeable improvements in F1 score. Moreover, MAE is reduced by 15.1% and 19.0%, respectively, with similarly significant reductions observed in SAE. These results further underscore the effectiveness of the LAB architecture in capturing essential appliance-specific characteristics.

For appliances such as the dishwasher, washing machine, and microwave, the proposed model continues to deliver consistent performance improvements, aligning with the results observed in Scenario 1 on the UK-DALE dataset, and therefore will not be elaborated further. The discrete abstraction mechanism introduced in the LAB module contributes to improved magnitude stability, thereby reducing amplitude-related errors such as MAE and SAE. Meanwhile, the global information compensation mechanism promotes waveform smoothing and suppresses spurious fluctuations, which helps reduce misclassification events and supports improved F1 performance. Furthermore, appliance-specific load patterns, such as burst-type versus steady-state behavior, high-power versus low-power consumption, and sparse versus frequent switching, exhibit different sensitivities to evaluation metrics. As a result, the observed performance variations reflect the differing emphases of the metrics rather than limitations in model stability. The proposed model demonstrates notable advantages in magnitude stability and cross-domain robustness, which explains its superior performance on MAE and SAE. This also explains why, for certain appliances, the proposed model may achieve slightly lower F1 scores while obtaining the best MAE and SAE results.

**Fig. 4:**
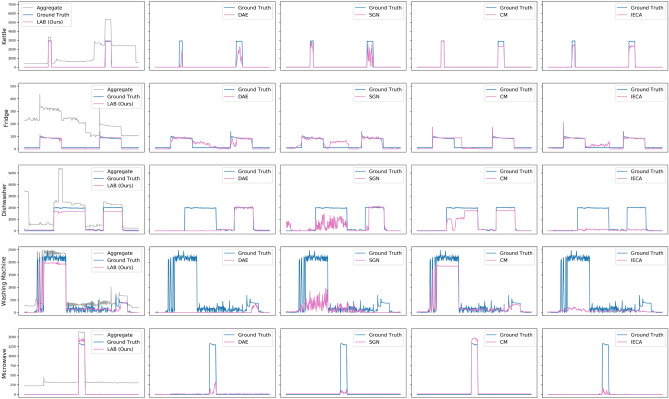
Results of five target appliance power disaggregation for Scenario 1 on the UK-DALE dataset. From top to bottom, the appliances are the kettle, fridge, dishwasher, washing machine, and microwave. The vertical axis represents power (in watts, W), and the horizontal axis represents time.

**Fig. 5:**
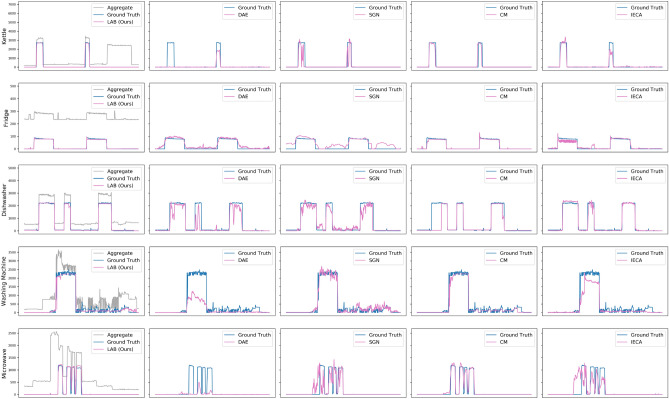
Results of five target appliance power disaggregation for Scenario 2 on the REFIT dataset. From top to bottom, the appliances are the kettle, fridge, dishwasher, washing machine, and microwave. The vertical axis represents power (in watts, W), and the horizontal axis represents time.

The disaggregation curves for both scenarios are shown in Fig. 4 and Fig. 5. In Scenario 1, the proposed model demonstrates a strong capability to closely fit the ground-truth signals. In contrast, other models exhibit common issues, such as missed detections, particularly for appliances with more complex or irregular usage patterns. In Scenario 2, although all models benefit from increased data diversity and volume, resulting in improved performance, their reconstructed signals still exhibit noticeable fluctuations. In contrast, the proposed model produces stable and accurate reconstructions, attributable to its enhanced representational capacity derived from the LAB module.

### Cross-dataset evaluation in Scenario 3

In Scenario 3, the proposed model continues to demonstrate strong performance. For the kettle and fridge, all models experience a slight performance degradation, which can be attributed to the distributional differences between the two datasets. Nevertheless, the proposed model consistently achieves superior performance across all evaluation metrics. It is worth noting the behavior observed for the remaining three appliances. For the dishwasher, washing machine, and microwave, many metrics even show slight improvements. This phenomenon can be attributed to the REFIT dataset providing a richer set of appliance instances, thereby alleviating the limitations of the UK-DALE dataset.

Averaged across all appliances, the proposed model achieves the best performance. It improves the F1 score by 1.4% over the second-best model, and reduces MAE and SAE by 6.3% and 8.3%, respectively. The comparative experiments across the three scenarios collectively demonstrate the strong generalization capability of the proposed model. It can perform accurate load disaggregation for unseen households.

### Ablation study

**Fig. 6:**
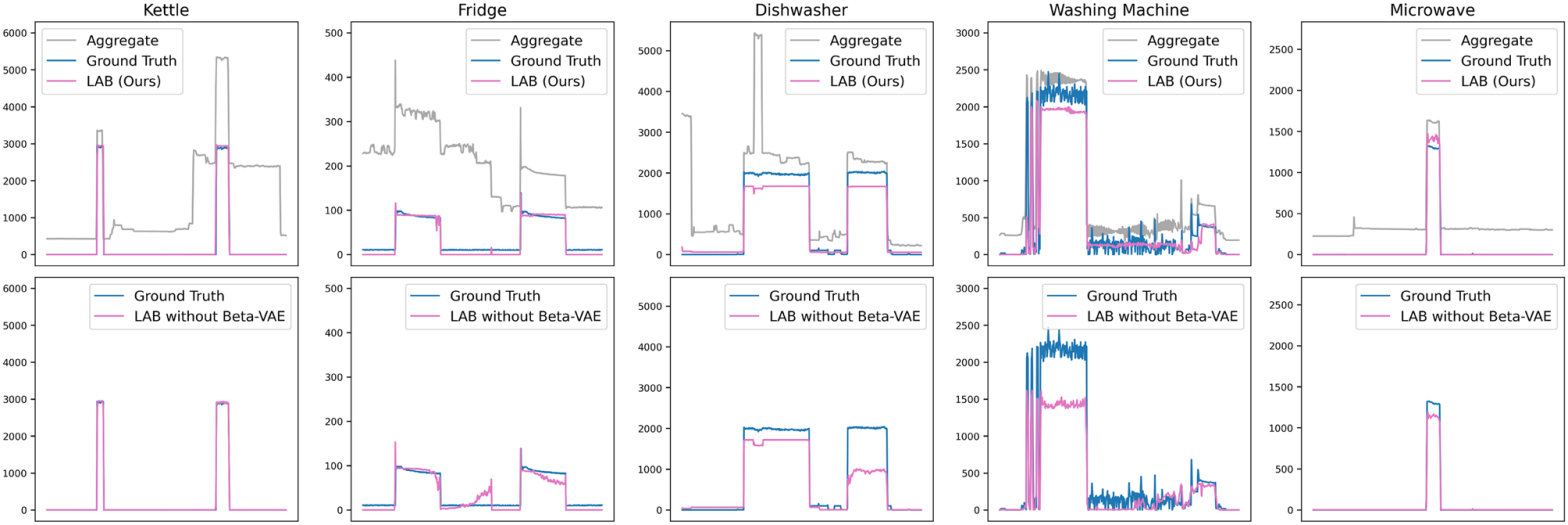
Results of LAB and LAB without Beta-VAE Structure on Scenario 1. The vertical axis represents power (in watts, W), and the horizontal axis represents time.

In the ablation study, the global information provided by the Beta-VAE component within the LAB module is removed, and the resulting modified model is compared to the full architecture under Scenario 1. Table 7 presents the $\Delta$ values of the evaluation metrics after the removal of the Beta-VAE component, while Fig. 6 illustrates the corresponding reconstructed signal curves. The results show that incorporating global information yields consistent improvements across most metrics, albeit with slight degradations in a few cases. This confirms that supplementing the LAB module with interpolated global features, while suppressing unnecessary local details, substantially enhances the model’s overall performance.

More specifically, only minor degradations were observed in certain metrics: SAE increased by 3.8% for the fridge, and MAE and SAE increased by 4.2% and 16.1%, respectively, for the microwave. All other metrics showed clear improvements. These findings emphasize the importance of integrating global context into the latent representation for improving disaggregation stability and accuracy.

**Table 7:** LAB without Beta-VAE Structure.

Appliance	$\Delta$F1 score	$\Delta$MAE (W)	$\Delta$SAE (W)
Kettle	−0.0131	+0.9154	+1.0249
Fridge	−0.0329	+2.0324	−0.3269
Dishwasher	−0.2039	+12.8546	+14.7916
Washing Machine	−0.4581	+4.5832	+4.8486
Microwave	−0.0874	−0.3759	−1.3271
Average	−0.1591	+4.0020	+3.8022

### Hyperparameter selection

#### temperature parameter

**Fig. 7:**
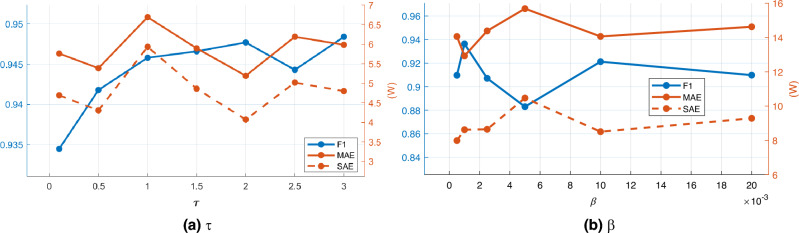
Different values of hyperparameters correspond to changes in model metrics.

Fig. 7a illustrates the impact of the temperature parameter $\tau$ used in soft quantization of the codebook, using the kettle appliance in Scenario 1 as an example. The optimal value of this critical hyperparameter was determined through a series of controlled experiments. When $\tau$ increases from 0.1 to 3.0, the F1 score shows an overall upward trend, reaching its highest and second-highest values at $\tau = 3.0$ and $\tau = 2.0$, respectively. However, both MAE and SAE decrease as $\tau$ increases up to 2.0, and begin to rise when $\tau$ exceeds this threshold. Considering the trade-off across all three metrics, $\tau = 2.0$ is selected as the most appropriate value for the kettle appliance.

This behavior is attributed to the influence of $\tau$ on the degree of soft quantization, which affects the balance between discrete and smooth representations in the latent space. When $\tau$ is small, the model tends to rely more heavily on a single codebook vector, resulting in sharper and more discrete representations. In contrast, larger values of $\tau$ encourage the model to incorporate contributions from multiple codebook vectors, leading to smoother latent representations. This variation highlights the sensitivity of model performance to the soft quantization behavior controlled by $\tau$. It suggests that different appliances may benefit from appliance-specific tuning of $\tau$ to optimize disaggregation performance.

#### $\beta$ in Beta-VAE

Fig. 7b illustrates the impact of the hyperparameter $\beta$ in Beta-VAE, with the fridge appliance under Scenario 1 as an example. The experiments show that, in the proposed model, setting $\beta$ to a small value facilitates stable convergence, whereas larger values tend to interfere with training. As $\beta$ varies from 0.0005 to 0.02, SAE exhibits only minor fluctuations, while MAE and F1 score show slight variations. When $\beta$ is set to 0.001, the F1 score reaches its highest value, and the MAE attains its minimum. Considering all three metrics comprehensively, $\beta =0.001$ is selected as the optimal setting for the experiments.

### Model size

Table 8 reports the model size of all compared methods. Since all models adopt an S2S architecture but differ in their sliding-window lengths, the computational cost is normalized using Per-Output Floating Point Operations (POFLOPs)^31^, defined as the average number of FLOPs needed to produce one output point.

**Table 8:** Model size and computational cost.

Model	Parameters (M)	POFLOPs (M)	CCS
LAB	3.52	3.22	3.31
DAE	16.82	0.06	5.09
SGN	59.74	0.34	18.16
CM	2.95	4.70	4.18
IECA	3.64	2.08	2.55

Since both the number of parameters and the FLOPs need to be jointly considered^32^, a combined complexity score (CCS) is introduced as a unified metric for assessing the overall computational complexity of a model by combining parameter count and computational cost. The CCS is defined as:

24 $$\begin{aligned} \text {CCS} = \alpha P + (1-\alpha ) F. \end{aligned}$$

Here, *P* denotes the total number of parameters of a model, and *F* denotes POFLOPs. $\alpha \in [0, 1]$ controls the relative importance of parameter size versus computational cost. In the study, $\alpha$ is set to 0.3, thereby placing greater emphasis on computational efficiency.

A lower CCS score indicates a smaller model size and reduced computational overhead. The proposed model achieves a CCS score of 3.31, which is only marginally higher than that of IECA, yet it attains the best generalization performance among all compared models. This demonstrates the superior efficiency of the proposed model.

### Latent space alignment analysis

#### Definition of latent generalizability

Latent generalizability is defined as the ability of a model to maintain class separability and structural stability in the latent space under distribution shift. Specifically, when the input distribution changes (e.g., across different houses), the geometric structure of the latent representations remains stable, such that samples belonging to the same appliance class remain clustered and inter-class boundaries are preserved. In other words, even if the statistical properties of the input signals vary, the latent distribution does not exhibit significant collapse, drift, or distortion that would compromise semantic separability.

In the proposed LAB framework, the SoftVQ-VAE component enhances the structural regularity and stability of latent representations through soft vector quantization and regularization constraints. The Beta-VAE module further improves representation robustness via an information bottleneck mechanism that suppresses redundant and noise-sensitive features while preserving globally informative patterns. This encourages the model to focus on appliance-specific characteristics rather than domain-specific variations. Moreover, the scoring network integrates local abstractions with global continuous representations, enabling a balanced interaction between fine-grained feature encoding and global semantic abstraction. Overall, the design is theoretically grounded in established principles of representation learning. The supplementary material provides a detailed mathematical formulation in Section “Mathematical Formulation”.

#### Visualization of latent space

**Fig. 8:**
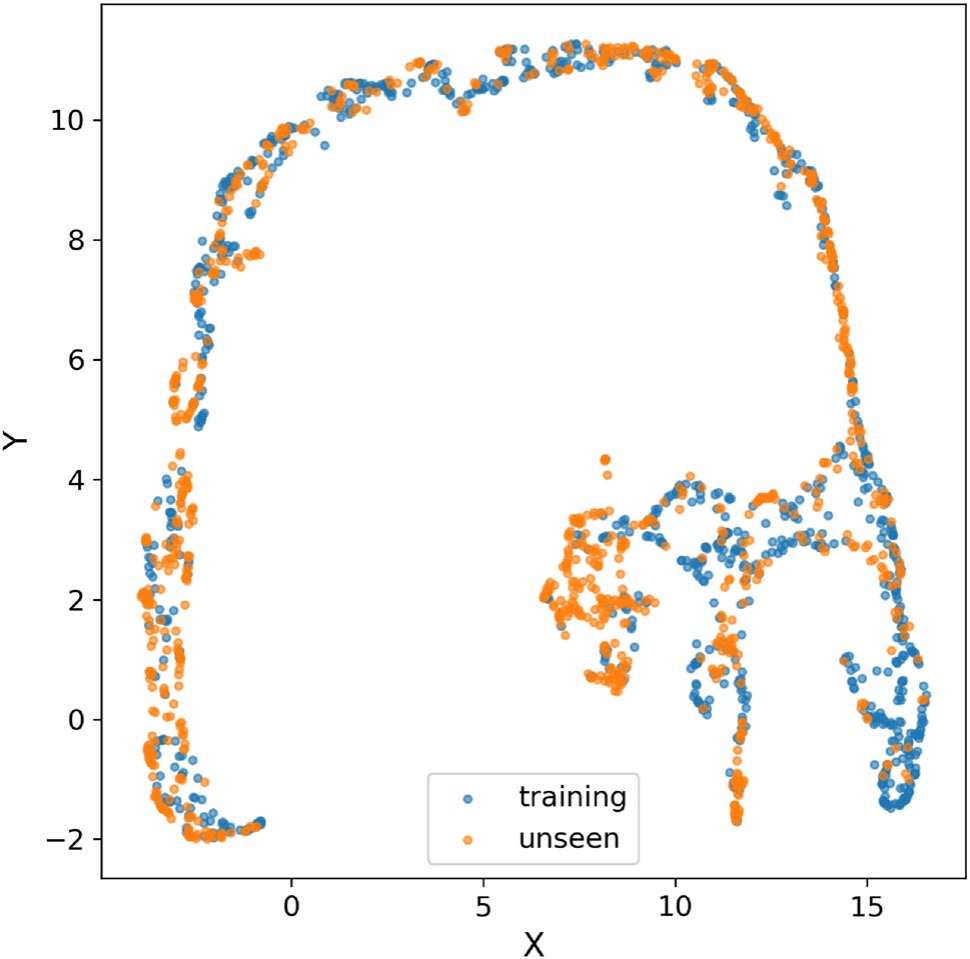
UMAP visualization of the kettle’s latent embeddings. Blue points represent samples from training houses, while orange points represent samples from unseen houses. The substantial overlap and consistent geometric structure between the two distributions indicate effective cross-domain alignment and structural stability of the learned latent representations.

Uniform Manifold Approximation and Projection (UMAP) is a nonlinear dimensionality reduction technique that projects high-dimensional representations into a low-dimensional space while preserving local neighborhood structure. By maintaining intrinsic geometric relationships among samples, UMAP enables intuitive visualization of the distribution and relative alignment of latent representations across different domains.

The latent embeddings produced by the LAB module are visualized using UMAP for samples from both training and unseen houses. As illustrated in Fig. 8 (using the kettle as a representative example), the embeddings from the two domains exhibit substantial overlap in the projected space, with consistent global structure and no evident domain-specific separation. This observation suggests effective cross-domain alignment and structural stability in the learned latent representations.

Similar patterns are observed across other appliances, indicating that the alignment effect is not appliance-specific. The visualizations for the remaining appliances are provided in Fig. S1 in the supplementary material.

#### Domain shift analysis

Maximum Mean Discrepancy (MMD) is a kernel-based statistical measure used to quantify the discrepancy between two probability distributions. Given samples drawn from two domains, MMD computes the distance between their mean embeddings in a reproducing kernel Hilbert space (RKHS). Formally, for two distributions *P* and *Q*, the squared MMD is defined as:

25 $$\begin{aligned} \text {MMD}^2(P,Q)=\Vert \mathbb {E}_{x\sim P}[\phi (x)]-\mathbb {E}_{y\sim Q}[\phi (y)]\Vert _{\mathscr {H}}^2, \end{aligned}$$

where $\phi (\cdot )$ denotes a feature mapping into an RKHS. In practice, the metric is estimated using minibatch samples and a Gaussian kernel function.

Since LAB, CM, and IECA adopt encoder–decoder architectures, the encoder outputs are used as latent representations for domain discrepancy analysis. In contrast, DAE and SGN do not follow an explicit encoder–decoder structure; therefore, feature representations are extracted from their key convolutional layers, which capture high-level semantic information and serve as comparable latent embeddings. A large number of samples from different appliances are randomly selected to ensure statistical reliability. $\text {MMD}^2$ is then computed between the training and unseen houses for each model. The resulting $\text {MMD}^2$ values for LAB, CM, and IECA are $9.58 \times 10^{-6}$, $4.15 \times 10^{-2}$, and $3.49 \times 10^{-3}$, respectively, while those for DAE and SGN are $1.23 \times 10^{-3}$ and $4.79 \times 10^{-4}$. Notably, the $\text {MMD}^2$ value of LAB is several orders of magnitude lower than those of the other models, suggesting that LAB effectively mitigates domain-induced distribution shift at the representation level. Detailed $\text {MMD}^2$ evaluation results are provided in Table S1 of the supplementary material.

## Conclusion

This work addresses the generalization challenges in NILM by proposing a novel hybrid model, termed LAB Transformer, built upon an S2S framework. The model integrates convolutional and Transformer architectures with an embedding-constrained generative module that combines discrete local features with global contextual information. This design enables refinement and re-encoding of encoder outputs to produce more generalizable and discriminative latent representations. A linear interpolation mechanism is introduced to reduce model size and inference time during global feature extraction. By leveraging this hybrid architecture, the model achieves significant improvements in generalization, particularly for complex multi-state appliances.

Experimental evaluations on two real-world datasets, UK-DALE and REFIT, show that the proposed model outperforms four state-of-the-art baselines across multiple evaluation metrics. The model consistently achieves superior F1 scores and delivers average reductions of 22.8% in MAE and 24.7% in SAE, demonstrating its effectiveness in both signal reconstruction and appliance stat detection. These gains are observed across diverse appliances, including both frequently used devices, such as the fridge and kettle, and complex appliances, such as the dishwasher and washing machine, even in the presence of noise and data imbalance. The cross-dataset evaluation further confirms the robust generalization ability of the proposed model. This work contributes toward the development of more robust and scalable NILM systems for real-world smart energy applications. In future work, we aim to explore model lightweighting and compression techniques to further enhance the practicality of our method for deployment in resource-constrained environments.

## Supplementary Information

Supplementary Information.

## Data Availability

The data used in this study were obtained from the publicly available UK-DALE and REFIT datasets, which are accessible via the repositories cited in DOI: 10.1038/sdata.2015.7 and DOI: 10.1038/sdata.2016.122, respectively.
