# Supplementary material for: Latent abstraction bridge transformer for generalizable nonintrusive load monitoring
Source: Sci Rep. 2026 Apr 30;16:20046. doi: 10.1038/s41598-026-48516-0 (PMC13319445; doi:10.1038/s41598-026-48516-0)
Supplement: Supplementary file 1 — Supplementary Information. [file 41598_2026_48516_MOESM1_ESM.pdf]

Supplementary Material: Latent Abstraction Bridge Transformer for Generalizable Nonintrusive Load Monitoring

Yuan Liu[1], Zhengmin Kong[1,*], Tao Huang[2], Yang Yang[1], Chenjie Song[1], Qing-Long Han[3], and Boyang Huang[4]

[1]Wuhan University, School of Electrical Engineering and Automation, Department of Artificial Intelligence and Automation, Wuhan, 430072, China
[2]James Cook University, College of Science and Engineering, and Centre for AI and Data Science Innovation, Cairns, QLD, 4878, Australia
[3]Swinburne University of Technology, School of Engineering, Melbourne, VIC, 3122, Australia
[4]Electric Power Research Institute of China Southern Power Grid, and also with Guangdong Provincial Key Laboratory of Intelligent Measurement and Advanced Metering of Power Grid, Guangzhou,510700, China
[*]corresponding.author: zmkong@whu.edu.cn

ABSTRACT

Nonintrusive load monitoring (NILM) is an effective approach for energy management that disaggregates the total power measured at the main power inlet into appliance-level power signals. NILM algorithms have achieved remarkable progress in recent years. However, accurately reconstructing appliance-level power signals from unseen, complex, and diverse aggregated data remains a formidable challenge. To address this challenge, this article proposes a novel hybrid load disaggregation model, the Latent Abstraction Bridge (LAB) Transformer, built on a sequence-to-sequence (S2S) framework that integrates a convolutional neural network (CNN) and a Transformer architecture with an embedding-constrained generative network termed LAB. The LAB effectively balances local discrete details and global information by leveraging a soft vector-quantized variational autoencoder (SoftVQ-VAE) and a beta-variational autoencoder (Beta-VAE) to constrain the encoder's output representations, thereby considerably improving the model's ability to generalize and discriminate in latent space. Moreover, we use parameter-free linear interpolation to recover the lengths of Beta-VAE output vectors, preserving essential global information while suppressing unnecessary local details, thereby substantially reducing the parameter count. The effectiveness of the proposed model is validated on two datasets: UK-DALE and REFIT. Experimental results indicate that it achieves the best F1 score, while lowering the mean absolute error (MAE) and signal aggregation error (SAE) by 22.8% and 24.7%, respectively, compared to several recent state-of-the-art models.
keywords:Beta-variational autoencoder, Soft vector-quantized variational autoencoder, Latent abstraction bridge (LAB), Transformer, Nonintrusive load monitoring

LAB Forward Procedure

Algorithm 1: LAB Forward Procedure

Input: Encoded feature sequence $Y_{\text{out}} \in \mathbb{R}^{B\times L\times D}$
Output: LAB output $Y_{\text{LAB}} \in \mathbb{R}^{B\times L\times D}$

`// --- Beta-VAE ---`
$h \leftarrow \text{MeanPool[dim=1]}(Y_{\text{out}})$; ▷ $[B,D]$
$\mu \leftarrow \text{Linear}(h)$; ▷ $[B,D]$
$\log\sigma^2 \leftarrow \text{Linear}(h)$; ▷ $[B,D]$
$\varepsilon \sim \mathcal{N}(0,I)$;
$z \leftarrow \mu + \varepsilon \odot \exp(0.5\log\sigma^2)$; ▷ $[B,D]$
`// global latent representation`
$z_g \leftarrow \text{Linear}(z)$; ▷ $[B, D\times L_0]$
$H_1 \leftarrow \text{Upsample}(z_g)$; ▷ `Interpolation,` $[B,D,L]$
`// --- SoftVQ-VAE ---`
$Z_e \leftarrow \text{Linear}(Y_{\text{out}})$; ▷ $[B,L,D]$
`// Compute similarity between` Z_e `and codebook` $E \in \mathbb{R}^{N\times D}$
$W \leftarrow \text{Softmax}(-\text{distance}(Z_e, E))$; ▷ $[B,L,N]$
$Z_q \leftarrow W \cdot E$; ▷ $[B,L,D]$
$H_2 \leftarrow Z_q$; ▷ $[B,D,L]$
`// --- Scoring network ---`
$s_1 \leftarrow \text{Score}(H_1)$; ▷ $[B,1,L]$
$s_2 \leftarrow \text{Score}(H_2)$; ▷ $[B,1,L]$
$\alpha \leftarrow \text{Softmax}([s_1, s_2])$; ▷ $[B,2,L]$
$H \leftarrow \alpha_1 \odot H_1 + \alpha_2 \odot H_2$; ▷ $[B,D,L]$
`// --- Output ---`
$Y_{\text{LAB}} \leftarrow H$; ▷ $[B,L,D]$
return Y_{LAB};

The forward process of the proposed LAB module is summarized in Algorithm 1. Given the encoded feature sequence $Y_{\text{out}} \in \mathbb{R}^{B\times L\times D}$, the LAB module first extracts latent features through the Beta-VAE branch and the SoftVQ-VAE branch, respectively. The resulting representations are then evaluated by the scoring network, which adaptively fuses the features to produce the final output.

In the Beta-VAE branch, the temporal features are first aggregated through mean pooling to obtain h. Two linear layers are then applied to estimate the parameters of the latent Gaussian distribution, namely the mean μ and the log-variance $\log\sigma^2$. To enable gradient backpropagation during stochastic sampling, the reparameterization trick is adopted. Specifically, a noise variable $\varepsilon \sim \mathcal{N}(0,I)$ is sampled, and the latent variable is computed as $z = \mu + \varepsilon \odot \exp(0.5\log\sigma^2)$. The sampled latent vector is then projected through a linear layer and expanded via interpolation to obtain the global latent feature H_1.

In the SoftVQ-VAE branch, Y_{out} is first projected through a linear layer to obtain Z_e. A learnable codebook $E \in \mathbb{R}^{N\times D}$ is then introduced to represent a set of prototype embeddings. The similarity between Z_e and each codebook entry is computed, and a softmax operation is applied to obtain the assignment weight matrix W. Based on these weights, a weighted combination of the codebook vectors is performed to generate the quantized representation Z_q. Finally, the quantized features are reshaped to produce the latent representation H_2.

The scoring network takes H_1 and H_2 as inputs and produces temporal importance scores s_1 and s_2, respectively. These scores are concatenated and normalized using a softmax operation to obtain the fusion weights α_1 and α_2. Since the weights are computed along the channel dimension, they are further replicated along the channel dimension so that their size becomes $[B,D,L]$ to match the feature dimensions of H_1 and H_2. Finally, the two representations are fused through element-wise weighted combination, yielding the fused feature H, which is taken as the output of the LAB module, i.e., Y_{LAB}.

Visualization of Latent Space

Fig. S1 presents dimensionality-reduced visualizations of the latent embeddings for the remaining appliances, excluding the kettle. The data points from training and unseen houses exhibit substantial overlap and consistent geometric structure in the

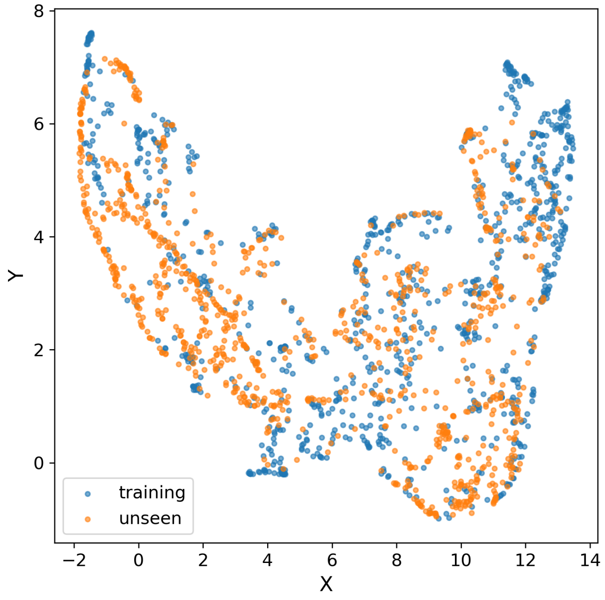

(a) Fridge

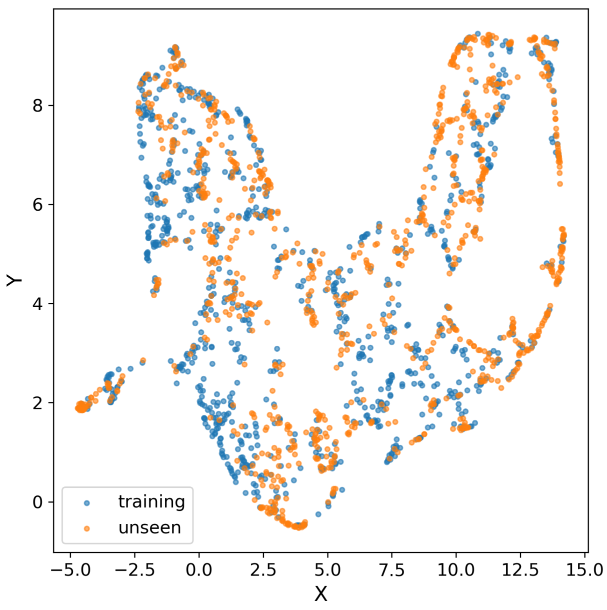

(b) Dishwasher

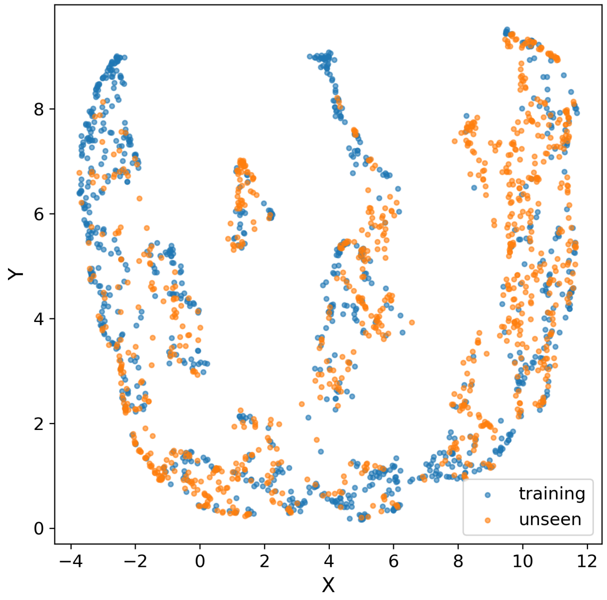

(c) Washing Machine

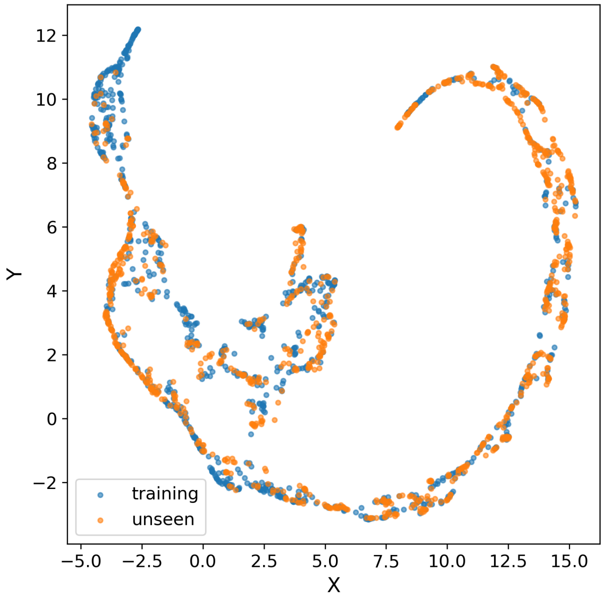

(d) Microwave

Figure S1. UMAP visualization of latent embeddings for different appliances. Blue points represent samples from training houses, while orange points represent samples from unseen houses. The substantial overlap and consistent geometric structure across domains indicate effective cross-domain alignment and structural stability of the learned latent representations.

projected space, without evident collapse or distortion. These observations provide intuitive evidence of effective cross-domain alignment in the learned latent representations.

Domain Shift Analysis

Table S1 reports the MMD^2 values computed for all compared models. Since LAB, CM, and IECA adopt encoder–decoder architectures, the encoder outputs are used as latent representations for domain discrepancy analysis. In contrast, DAE and SGN do not follow an explicit encoder–decoder structure. Therefore, feature representations are extracted from their key convolutional layers, which capture high-level semantic information and serve as comparable latent embeddings. A large number of samples from different appliances are randomly selected to ensure statistical reliability. MMD^2 is then computed between the training and unseen houses for each model. Although the proposed model achieves values several orders of magnitude lower than those of the other methods, architectural differences across models imply that the selected latent representations used for MMD computation may not be perfectly equivalent. Therefore, the results should be interpreted as indicative rather than definitive, and primarily serve to support the evaluation of domain shift reduction.

Table S1. The MMD^2 Values Computed for All Models

Model	Kettle	Fridge	Dishwasher	Washing Machine	Microwave	Average
**LAB (Ours)**	1.35×10^{-6}	3.09×10^{-5}	1.18×10^{-7}	4.52×10^{-6}	1.10×10^{-5}	9.58×10^{-6}
DAE [11]	1.18×10^{-4}	1.27×10^{-5}	2.50×10^{-3}	7.29×10^{-5}	3.46×10^{-3}	1.23×10^{-3}
SGN [14]	5.94×10^{-6}	8.46×10^{-4}	1.48×10^{-5}	1.50×10^{-3}	3.20×10^{-5}	4.79×10^{-4}
CM [20]	2.40×10^{-2}	6.59×10^{-2}	3.09×10^{-2}	4.81×10^{-2}	3.87×10^{-2}	4.15×10^{-2}
IECA [9]	3.22×10^{-3}	3.68×10^{-3}	4.20×10^{-3}	2.70×10^{-3}	3.63×10^{-3}	3.49×10^{-3}

[9] Yu, H., Pang, C., Xuan, Y., Chen, Y. & Zeng, X. Sequence-to-sequence-based Beta-VAE combined with IECA attention mechanism for energy disaggregation algorithm. IEEE Transactions on Instrumentation Meas. 72, 1–13, DOI: 10.1109/TIM.2023.3325867 (2023).
[11] Kelly, J. & Knottenbelt, W. Neural NILM: Deep neural networks applied to energy disaggregation. In Proceedings of the 2nd ACM International Conference on Embedded Systems for Energy-Efficient Built Environments, BuildSys '15, 55–64, DOI: 10.1145/2821650.2821672 (Association for Computing Machinery, New York, NY, USA, 2015).
[14] Shin, C. et al. Subtask gated networks for non-intrusive load monitoring. In Proceedings of the AAAI Conference on Artificial Intelligence, vol. 33, 1150–1157, DOI: 10.1609/aaai.v33i01.33011150 (2019).
[20] Sun, Y., Feng, J., Yuan, L., Su, M. & Luan, W. Transfer learning for multiappliance-task nonintrusive load monitoring. IEEE Transactions on Instrumentation Meas. 74, 1–12, DOI: 10.1109/TIM.2025.3541652 (2025).

Performance Range

Tables S2 and S3 summarize the ranges of evaluation metrics for all compared models under multiple random seed settings. In contrast, the results reported in Table 6 of the main text correspond to the cross-dataset evaluation setting and are obtained by evaluating the best-performing checkpoints of each model under Scenario 2. Therefore, performance ranges are not provided for this case, as the evaluation reflects a deployment-oriented protocol in which the best-trained model is selected for real-world application [33].

Table S2. Performance Range Across Multiple Runs on Scenario 1

Metric	Model	Kettle	Fridge	Dishwasher	Washing Machine	Microwave
F1 score	**LAB (Ours)**	[0.940, 0.954]	[0.918, 0.946]	[0.603, 0.660]	[0.853, 0.911]	[0.695, 0.770]
	DAE	[0.749, 0.835]	[0.785, 0.840]	[0.550, 0.634]	[0.135, 0.222]	[0.296, 0.430]
	SGN	[0.902, 0.935]	[0.873, 0.906]	[0.112, 0.245]	[0.143, 0.192]	[0.300, 0.530]
	CM	[0.954, 0.974]	[0.921, 0.935]	[0.473, 0.523]	[0.897, 0.920]	[0.691, 0.713]
	IECA	[0.915, 0.947]	[0.890, 0.928]	[0.310, 0.371]	[0.203, 0.340]	[0.363, 0.413]
MAE (W)	**LAB (Ours)**	[5.00, 5.79]	[11.67, 14.10]	[19.23, 22.39]	[8.22, 9.04]	[8.12, 9.66]
	DAE	[9.05, 13.00]	[19.16, 22.23]	[25.37, 28.75]	[16.15, 20.52]	[12.60, 13.37]
	SGN	[17.70, 18.86]	[15.25, 23.78]	[26.14, 29.96]	[15.00, 16.24]	[19.67, 21.04]
	CM	[5.51, 6.07]	[11.78, 13.31]	[22.74, 26.43]	[19.61, 22.78]	[6.82, 8.36]
	IECA	[10.15, 14.54]	[13.39, 15.80]	[18.15, 33.44]	[14.81, 17.27]	[13.09, 15.41]
SAE (W)	**LAB (Ours)**	[3.80, 4.86]	[8.21, 8.91]	[15.90, 19.69]	[7.01, 8.26]	[7.37, 8.92]
	DAE	[7.49, 10.15]	[10.67, 12.09]	[23.78, 25.36]	[14.95, 18.62]	[9.21, 10.54]
	SGN	[13.03, 14.26]	[9.40, 13.30]	[22.72, 26.73]	[11.89, 13.22]	[16.04, 17.72]
	CM	[3.99, 4.78]	[7.86, 8.95]	[17.70, 23.92]	[18.70, 21.05]	[5.87, 6.45]
	IECA	[6.86, 10.45]	[7.26, 10.56]	[17.24, 28.83]	[12.93, 13.47]	[8.53, 12.38]

Table S3. Performance Range Across Multiple Runs on Scenario 2

Metric	Model	Kettle	Fridge	Dishwasher	Washing Machine	Microwave
F1 score	**LAB (Ours)**	[0.710, 0.749]	[0.830, 0.864]	[0.706, 0.734]	[0.763, 0.799]	[0.548, 0.661]
	DAE	[0.207, 0.310]	[0.771, 0.816]	[0.382, 0.482]	[0.266, 0.329]	[0.414, 0.449]
	SGN	[0.462, 0.522]	[0.756, 0.822]	[0.060, 0.119]	[0.539, 0.595]	[0.514, 0.592]
	CM	[0.694, 0.723]	[0.834, 0.850]	[0.669, 0.682]	[0.761, 0.795]	[0.494, 0.549]
	IECA	[0.427, 0.521]	[0.836, 0.875]	[0.273, 0.281]	[0.337, 0.419]	[0.501, 0.614]
MAE (W)	**LAB (Ours)**	[13.61, 16.76]	[10.43, 12.57]	[3.08, 4.23]	[9.38, 12.00]	[7.01, 8.70]
	DAE	[27.50, 31.79]	[19.04, 19.90]	[4.57, 6.76]	[26.30, 33.06]	[9.00, 9.52]
	SGN	[30.76, 35.40]	[22.44, 28.67]	[16.91, 24.31]	[29.74, 30.73]	[9.87, 10.23]
	CM	[16.58, 19.35]	[13.02, 14.88]	[6.09, 6.89]	[17.65, 21.78]	[6.00, 6.80]
	IECA	[27.63, 31.07]	[12.97, 14.73]	[6.10, 7.14]	[23.80, 26.37]	[10.49, 11.32]
SAE (W)	**LAB (Ours)**	[10.00, 11.61]	[7.03, 8.04]	[3.00, 3.22]	[8.22, 9.88]	[6.23, 6.72]
	DAE	[19.54, 24.01]	[8.95, 11.30]	[3.89, 4.77]	[25.18, 31.42]	[7.01, 7.42]
	SGN	[22.00, 26.73]	[12.29, 18.37]	[15.35, 21.42]	[25.24, 26.89]	[7.96, 8.64]
	CM	[12.59, 14.29]	[8.00, 8.85]	[5.24, 6.20]	[13.40, 16.92]	[5.43, 6.22]
	IECA	[16.16, 22.78]	[7.19, 8.06]	[3.30, 3.97]	[17.56, 20.98]	[6.89, 7.48]

[33] Lin, J., Ma, J., Zhu, J. & Liang, H. Deep domain adaptation for non-intrusive load monitoring based on a knowledge transfer learning network. IEEE Transactions on Smart Grid. 13, 280–292, DOI: 10.1109/TSG.2021.3115910 (2022).

Mathematical Formulation

The load disaggregation task is formulated as minimizing the MSE subject to two latent space constraints imposed by the Beta-VAE and SoftVQ-VAE branches, respectively [24] [27]. Formally, the optimization problem is defined as:

$$\min_{\theta,\phi} \; L = \frac{1}{BT}\sum_{b=1}^{B}\sum_{t=1}^{T}\|x_{b,t}-\hat{x}_{b,t}\|_2^2, \tag{S1}$$

$$\text{subject to} \quad \underbrace{D_{\mathrm{KL}}(q_\phi(z|x)\|p(z)) \le \varepsilon_1}_{\text{(C1) Distributional Regularity}}, \tag{S2}$$

$$\underbrace{D_{\mathrm{KL}}(q(e)\|U) \le \varepsilon_2}_{\text{(C2) Prototype Distribution Constraint}}. \tag{S3}$$

In the formulation, θ and ϕ denote the parameters of the decoder and encoder networks, respectively. The objective function L represents the MSE loss. Specifically, $x_{b,t}$ and $\hat{x}_{b,t}$ denote the true and reconstructed values at time step t of the b-th

sample in a mini-batch, respectively, while B and T represent the batch size and the sequence length. The first constraint, $D_{\mathrm{KL}}(q_\phi(z|x)\|p(z)) \leq \varepsilon_1$, enforces distributional regularity on the latent representation. Here, x represents an input sample, $q_\phi(z|x)$ denotes the approximate posterior distribution produced by the encoder, $p(z)$ denotes a predefined prior distribution in the latent space, which is typically assumed to be the Gaussian distribution. The constant ε_1 specifies the maximum allowable divergence between the posterior and the prior distribution. This constraint promotes a smooth, compact latent space, thereby improving the consistency of learned representations across varying input conditions. The second constraint, $D_{\mathrm{KL}}(q(e)\|U) \leq \varepsilon_2$, imposes a prototype distribution constraint on the codebook usage. In this expression, $q(e)$ denotes the empirical distribution of prototype assignments induced by the encoder, while U represents the uniform distribution over the prototype codebook. This constraint encourages balanced utilization of the prototype embeddings. The constant ε_2 controls the strength of this regularization.

To efficiently solve the constrained optimization problem, the method of Lagrange multipliers is adopted to relax it into an unconstrained objective, which is optimized during training as the following loss function:

$$\mathscr{L}_{\text{total}} = \mathscr{L}_{\text{rec}} + \beta \cdot \mathscr{L}_{\text{KL}} + \gamma \cdot \mathscr{L}_{\text{codebook}}, \tag{S4}$$

where β and γ act as Lagrange multipliers that control the trade-off between reconstruction fidelity and the satisfaction of the latent distribution constraints.

Next, we analyze the optimization process. For clarity, we first present the gradient propagation in the baseline model without the LAB module, followed by the model with LAB to illustrate its effect on training dynamics. For comparison, the gradient propagation is first analyzed for the network without the LAB module. The input to the decoder is denoted as X for notational convenience. And to streamline the notation in the following derivation, the model's predicted output sequence is denoted as y (corresponding to $\hat{x}_{b,t}$), and the ground truth target sequence is denoted as y^* (corresponding to $x_{b,t}$). The output of the decoder is given by:

$$y = \text{Decoder}(X). \tag{S5}$$

The reconstruction loss is defined as the MSE:

$$L = \mathscr{L}_{\text{rec}} = L_{\text{MSE}}(y, y^*). \tag{S6}$$

By applying the chain rule, the gradient of the loss with respect to the decoder input X is:

$$\nabla_X L = \frac{\partial L}{\partial y}\frac{\partial y}{\partial X}. \tag{S7}$$

Let $J_D = \frac{\partial y}{\partial X}$ denote the Jacobian matrix of the decoder. The gradient expression becomes:

$$\nabla_X L = J_D^T \nabla_y L. \tag{S8}$$

Taking the Euclidean norm on both sides and utilizing the consistency property of the induced operator norm (spectral norm):

$$\|\nabla_X L\|_2 = \|J_D^T \nabla_y L\|_2 \leq \|J_D^T\|_2 \cdot \|\nabla_y L\|_2. \tag{S9}$$

Since the spectral norm of a matrix is equal to that of its transpose ($\|J_D^T\|_2 = \|J_D\|_2$), this simplifies to:

$$\|\nabla_X L\|_2 \leq \|J_D\|_2 \cdot \|\nabla_y L\|_2. \tag{S10}$$

While for the model with LAB, the decoder input is obtained by fusing the outputs of the Beta-VAE branch and the SoftVQ-VAE branch. Let H_1 and H_2 denote the feature representations produced by the two branches, respectively. The LAB module performs a gated fusion of the two representations, and the resulting decoder input can be expressed as:

$$H = \alpha_1 H_1 + \alpha_2 H_2, \tag{S11}$$

where α_1 and α_2 denote the weights generated by the scoring network. These weights satisfy $\alpha_1 \geq 0, \alpha_2 \geq 0$, and $\alpha_1 + \alpha_2 = 1$. The decoder output is therefore given by:

$$y = \text{Decoder}(H). \tag{S12}$$

The reconstruction loss remains the MSE:

$$L = \mathscr{L}_{\text{rec}} = L_{\text{MSE}}(y, y^*). \tag{S13}$$

By applying the chain rule, the gradient of the loss with respect to H is:

$$\nabla_H L = \frac{\partial L}{\partial y}\frac{\partial y}{\partial H}. \tag{S14}$$

Let $J_D = \frac{\partial y}{\partial H}$ denote the Jacobian matrix of the decoder. The gradient can therefore be written as:

$$\nabla_H L = J_D^T \nabla_y L. \tag{S15}$$

Since the decoder input H is a weighted combination of H_1 and H_2, the gradients with respect to the two branch outputs become:

$$\nabla_{H_1} L = \alpha_1 \nabla_H L, \tag{S16}$$
$$\nabla_{H_2} L = \alpha_2 \nabla_H L. \tag{S17}$$

Taking the Euclidean norm of both sides yields:

$$\|\nabla_{H_1} L\|_2 = \alpha_1 \|\nabla_H L\|_2, \tag{S18}$$
$$\|\nabla_{H_2} L\|_2 = \alpha_2 \|\nabla_H L\|_2. \tag{S19}$$

Substituting the previous derived bound for $\nabla_H L$ leads to:

$$\|\nabla_{H_1} L\|_2 \leq \alpha_1 \|J_D\|_2 \|\nabla_y L\|_2, \tag{S20}$$
$$\|\nabla_{H_2} L\|_2 \leq \alpha_2 \|J_D\|_2 \|\nabla_y L\|_2. \tag{S21}$$

Because the fusion weights satisfy $\alpha_1 \geq 0, \alpha_2 \geq 0$, and $\alpha_1 + \alpha_2 = 1$, the gradients propagated through the two branches are scaled by the corresponding adaptive weights, in contrast to equation (S10). Therefore, the LAB module introduces an adaptive gradient modulation mechanism, where the gradient contributions from the Beta-VAE branch and the SoftVQ-VAE branch are dynamically balanced during back-propagation. This mechanism encourages complementary learning between global and prototype-based representations, leading to more structured latent abstractions.

Let X denote the input feature to the LAB module. The outputs of the two branches can be viewed as nonlinear transformations of X. By applying the chain rule, the gradient of the loss with respect to X can be written as

$$\nabla_X L = \frac{\partial H_1}{\partial X}\nabla_{H_1} L + \frac{\partial H_2}{\partial X}\nabla_{H_2} L. \tag{S22}$$

Let $J_1 = \frac{\partial H_1}{\partial X}$ and $J_2 = \frac{\partial H_2}{\partial X}$ denote the Jacobian matrices of the two branches. The gradient becomes

$$\nabla_X L = J_1^T \nabla_{H_1} L + J_2^T \nabla_{H_2} L. \tag{S23}$$

Taking the Euclidean norm on both sides and applying the triangle inequality yields

$$\|\nabla_X L\|_2 \leq \|J_1^T \nabla_{H_1} L\|_2 + \|J_2^T \nabla_{H_2} L\|_2. \tag{S24}$$

Using the consistency property of the spectral norm, the following bound is obtained

$$\|\nabla_X L\|_2 \leq \|J_1\|_2 \|\nabla_{H_1} L\|_2 + \|J_2\|_2 \|\nabla_{H_2} L\|_2. \tag{S25}$$

Substituting the previously derived bounds for the branch gradients leads to

$$\|\nabla_X L\|_2 \leq (\alpha_1 \|J_1\|_2 + \alpha_2 \|J_2\|_2) \|J_D\|_2 \|\nabla_y L\|_2. \tag{S26}$$

Because the fusion weights satisfy $\alpha_1 \geq 0, \alpha_2 \geq 0$, and $\alpha_1 + \alpha_2 = 1$,

$$\|\nabla_X L\|_2 \leq \max(\|J_1\|_2, \|J_2\|_2) \|J_D\|_2 \|\nabla_y L\|_2. \tag{S27}$$

For the two branches, both mappings are composed of standard neural network operators. Specifically, the Beta-VAE branch consists of mean pooling, linear projections, reparameterization, and linear interpolation-based upsampling layers, all of which are Lipschitz continuous with finite operator norms under bounded inputs. The stochastic sampling step does not introduce

additional gradient amplification, since the injected noise is independent of the input and only acts as a multiplicative factor in the reparameterization. For the SoftVQ-VAE branch, the operations include distance computation, softmax-based assignment, and codebook aggregation. The softmax function has a bounded Jacobian, and the weighted summation is controlled by the finite norm of the codebook embeddings, ensuring that the overall mapping remains Lipschitz continuous. The downstream mapping comprises self-attention, layer normalization, and feed-forward layers. Under the constraint of bounded inputs enforced by layer normalization, the self-attention mechanism and the feed-forward networks exhibit bounded local Lipschitz constants. Specifically, the softmax function ensures bounded attention weights, while the linear projections possess finite spectral norms. Moreover, for the MSE loss, the gradient with respect to the network output is exactly the prediction error, which remains bounded given finite targets and model outputs. Consequently, all terms on the right-hand side of equation (S27) are bounded. This implies that $\|\nabla_X L\|_2$ admits a finite upper bound, thereby ensuring stable gradient propagation and preventing explosion through the entire architecture. While bounded gradients do not directly guarantee generalization, they reduce sensitivity to input perturbations, which is a commonly used proxy for robustness under distribution shift.

The above derivation provides an upper bound on the gradient norm with respect to the input feature X, revealing that the gradient propagation through the LAB module is jointly controlled by the spectral norms of the branch Jacobians and the downstream mapping. From an optimization perspective, the result suggests that constraining the spectral norms of J_1 and J_2 can effectively regulate the gradient flow, thereby improving training robustness and convergence behavior [34]. From a representation learning perspective, such boundedness (S27) reduces the sensitivity of the model to input perturbations and distribution variations [35], which is commonly associated with improved robustness under domain shift. As a result, the learned latent representations tend to exhibit enhanced structural stability. The experimental results reported in the subsection "Domain Shift Analysis" (full results in the supplementary material, Table S1) demonstrate that the proposed model achieves the lowest MMD^2 value. This indicates that LAB effectively mitigates domain shift at the representation level, which is consistent with the theoretical analysis.

[24] Kingma, D. P. & Welling, M. Auto-encoding variational bayes. arXiv preprint arXiv:1312.6114 (2013).

[27] Chen, H. et al. SoftVQ-VAE: Efficient 1-dimensional continuous tokenizer. In Proceedings of the Computer Vision and Pattern Recognition Conference (CVPR), 28358–28370 (2025).

[34] Miyato, T. et al. Spectral normalization for generative adversarial networks. arXiv preprint arXiv:1802.05957 (2018).

[35] Sokolić, J. et al. Robust large margin deep neural networks. IEEE Transactions on Signal Processing 65, 4265–4280 (2017).
